# A high sucrose detection threshold is associated with increased energy intake and improved post-prandial glucose response independent of the sweetness intensity of isocaloric sucrose solutions

**DOI:** 10.1038/s44324-023-00003-0

**Published:** 2024-01-29

**Authors:** Verena Preinfalk, Kerstin Schweiger, Leonie Hüller, Andreas Dunkel, Isabella Kimmeswenger, Corinna M. Deck, Petra Rust, Veronika Somoza, Gerhard E. Krammer, Jakob P. Ley, Barbara Lieder

**Affiliations:** 1 Christian Doppler Laboratory for Taste Research, Faculty of Chemistry, University of Vienna, Vienna, Austria; 2 Vienna Doctoral School in Chemistry (DoSChem), University of Vienna, Vienna, Austria; 3 Institute of Physiological Chemistry, Faculty of Chemistry, University of Vienna, Vienna, Austria; 4 Leibniz Institute for Food Systems Biology at the Technical University of Munich, Freising, Germany; 5 Department of Nutritional Science, Faculty of Life Sciences, University of Vienna, Vienna, Austria; 6 Symrise AG, Holzminden, Germany; 7 Institute of Clinical Nutrition, University of Hohenheim, Stuttgart, Germany

**Keywords:** Endocrine system and metabolic diseases, Metabolism

## Abstract

Several studies proposed a role for the sweet taste receptor in energy intake and blood glucose regulation, but little is yet known about the impact of the individual sweet taste perception. Here, we found in a cross-over human intervention study with 29 male participants that modulating the sweetness of an isocaloric sucrose solution did not influence postprandial plasma concentrations of blood glucose and associated hormones over 120 min and 2 h post-load energy intake. Independent of the sweetness of the test solution, tests persons with a higher sucrose detection threshold had an average of 402 ± 78.8 kcal (39 ± 21%) higher energy intake and a higher glucose/insulin ratio, combined with a higher liking for sweet tasting food, than the test persons of the low threshold group. The body composition suggested a higher fat-free mass in the high threshold group that may have influenced energy intake and post-prandial glucose responses.

## Introduction

Gustation, the taste sensation, is a key driver of food intake as it determines food choices, although its role in food intake behavior, satiety and energy balance on a mechanistic level is still poorly understood^1–4^.

Especially sweet taste has a powerful hedonic appeal and it has been proposed that sweet sensitivity correlates with sweet food liking. Higher sensitivities for sweetness, meaning lower sweet taste thresholds, have been associated with a decreased preference for sweet taste in healthy adults^5^ and children^6^. In contrast, Bossola et al. observed a positive correlation between sweet sensitivity and preference^7^. Despite these discrepancies, sweet liking is collectively assumed as a potential driver for weight gain^8^. In fact, the global consumption of sugars, like sucrose, fructose, and glucose, primarily in the form of sugar-sweetened beverages and foods, has increased over the past decades, paralleling the rise in obesity rates^9^. Recent data indicate prevalence of obesity is still rising, although the intake of sugars and sugar-sweetened beverages is declining^10,11^. Meta analyses of controlled trials and systematic reviews indicate that simple sugars do not behave differently from other macronutrients in driving weight gain^12^. But it should be considered that especially sugar sweetened beverages are less filling and induce poor energy compensation compared to solid foods^13^. Thus, sugar sweetened beverages are still associated with adverse effects^14^ and preferences for foods and beverages high in sugar are regarded as important contributors of body weight gain and the development of obesity and its co-morbidities^14,15^. With that, one identified key factor for the progression of obesity is an over-consumption of beverages high in added sugar^16^.

One strategy to reduce the calorie intake from sugar-sweetened beverages that still provide the palatable sweet taste is the usage of high intensity sweeteners with no or reduced calories. However, there is controversy about the metabolic health effects of sweet tasting compounds, especially non-caloric sweeteners. It has been proposed that not only sweet tasting carbohydrates contribute to insulin secretion and the regulation of blood glucose levels. Also, the perceived sweetness from non-caloric sweeteners has been hypothesized to modulate the cephalic phase insulin release (CIPR)^17–20^. A recent review by the WHO concluded that the short-term use of non-nutritive sweeteners (NNS) results in a small reduction in body weight and BMI in adults without any significant impact on cardiometabolic health, like fasting glucose, insulin, blood lipids, and blood pressure^21^. In contrast, the results obtained from prospective cohort studies suggested that higher NNS intake is associated with a higher body weight, increased risk of type 2 diabetes, and cardiovascular diseases. However, the evidence for adverse health effects of NNS is inconclusive, as the discrepancy between the results of randomized control trials and prospective cohort studies could be due to reverse causation^21^. Thus, there is a strong need for an improved understanding of the metabolic health effects of individual sweeteners.

The finding that the sweet taste receptor, consisting of the two subunits TAS1R2/TAS1R3, is not only present in the oral cavity, but also in extraoral tissues, such as the gastrointestinal tract, fueled the discussion about the involvement of the sweet taste receptor in nutrient sensing of carbohydrates and the corresponding physiological responses. Since the sweet taste receptor is activated not only by sweet tasting carbohydrates, the metabolic effects of non-caloric sweeteners based on chemoreceptor activation remain highly debated^22^. For example, the activation of sweet taste receptor in the oral cavity with a 10 mM saccharin solution for 45 s led to a small, but significant increase in cephalic phase insulin by 12% without affecting blood glucose concentrations^23^. On the other hand, results from a preceding human intervention study of our own group, in which the sweetness of isocaloric glucose and sucrose solutions were adjusted with the TAS1R3-antagonist lactisole, indicated that the type of the carbohydrate had a stronger impact on the regulation of blood glucose levels than the perceived sweetness^24^. This is in accordance with a finding from Dalenberg et al., showing that only the combination of the non-caloric sweetener sucralose and the carbohydrate maltodextrin, but not sucralose alone, impaired insulin sensitivity^25^. Furthermore, sucralose increased GLP-1 levels by 29.6% in overweight test persons^26^ and lead to a trend (*p* = 0.08) towards higher GIP concentrations^27^ in obese test persons, suggesting that sucralose intake could promote insulin resistance^28^. However, the role of the sweet taste of sucralose remained unclear in both studies. In addition to GLP-1, also the secretion of other satiety hormones, like serotonin, has been investigated in respect to the sweet taste receptor activation. A cell culture study showed that caloric as well as non-caloric sweeteners induce serotonin secretion in a human gastric parietal cell line via targeting TAS1R3^29^. Results from human intervention trials using the sweet taste inhibitor lactisole have proposed a role for TAS1R3 in secretion of the satiety hormones serotonin, GLP-1 and PYY, thereby contributing to the regulation of energy intake^30–32^.

Although the role of sweet taste receptor activation in the regulation of metabolic functions has been addressed in previous studies, only little is known about the impact of the individual sweet taste perception and the sweet taste sensitivity. Sweet taste perception is known to be affected by different variables like age, sex, body mass index (BMI), smoking, alcohol consumption, obesity, and type 2 diabetes mellitus (T2DM)^33^. The effect of body weight, mostly determined as the BMI, on sucrose taste thresholds is discussed controversially in the literature. On the one hand, it is described that subjects with a higher BMI had a higher sucrose detection threshold^34^, whereas other studies found the contrary association^35,36^, or no significant difference^37^. Higher sweet taste thresholds have been associated with higher blood glucose levels and therefore with T2DM^33^.

To summarize, several studies propose a role for the sweet taste receptor in energy intake and blood glucose regulation. However, the role of the individual sweet taste perception remains unclear. We hypothesized that individuals with a higher sensitivity towards sweet taste stimuli will show a stronger response by satiety hormones after receiving the sweeter test solution compared to the less sweet test solution.

Thus, in this study, we investigated the influence of different sweetness levels of an isocaloric sucrose solution on blood glucose regulation and energy intake in healthy male test persons in dependence of their sweet taste sensitivity. In detail, the sweetness of a 10% sucrose solution was modulated using either the steviol glycoside rebaudioside M (RebM, increased sweetness^38^), or lactisole (reduced sweetness^39^), in application relevant amounts in a randomized cross-over design. In addition, the individually perceived sweetness of the test solution, as well as the sucrose detection threshold beside the body constitution and food preferences were taken into consideration.

## Results

### Test persons with a high sucrose threshold showed a poorer discrimination of the test solutions

**Fig. 1:**
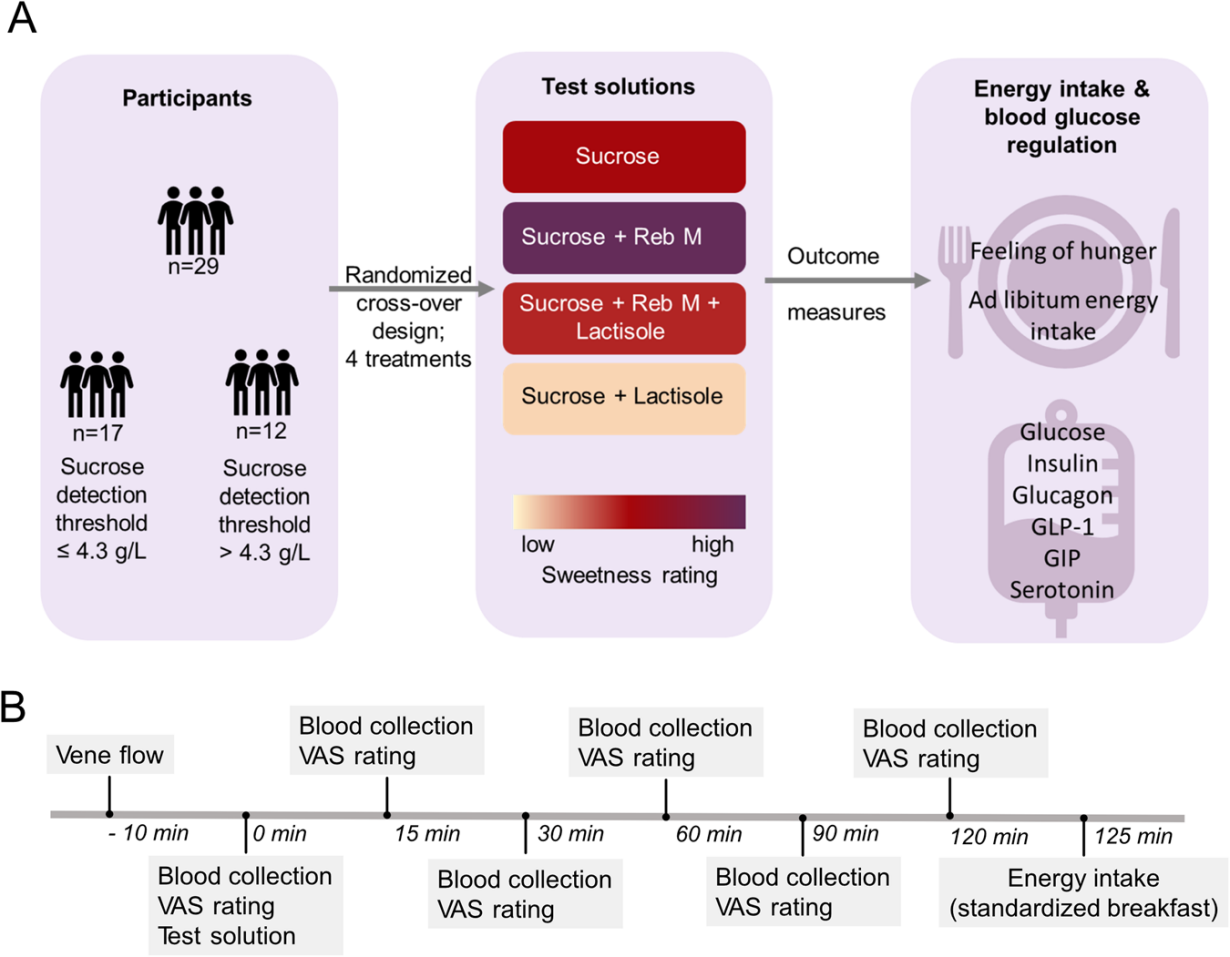
Overview of the study design and time-line of a study day. **A** In a four-armed, randomized cross-over intervention trial, 300 mL of a 10% sucrose solution alone or in combination with the sweet taste modulators rebaudioside M (30 mg/L, increased sweetness) or lactisole (15 mg/L, decreased sweetness) was given to 29 healthy young men. The participants were split into a high sweet taste threshold group (sucrose detection threshold ≤ 4.3 g/L) and a low sweet taste threshold group (sucrose detection threshold > 4.3 g/L). The metabolic response was analyzed over a time span of 120 min. **B** Time-line of study day for one treatment of the cross-over intervention trial comprising the four treatments displayed in **A**. VAS visual analogue scale.

To clarify the impact of the sweet taste perception on markers of blood glucose regulation and energy intake, we performed a cross-over human intervention study with four isocaloric 10% sucrose-based solutions with modulated sweetness (Fig. 1A). The test persons consumed 300 mL of the test solution and their postprandial plasma concentrations of blood glucose and associated hormones were monitored at six time points over a time span of 120 min as well as the 2 h post-load energy intake from a standardized breakfast (Fig. 1B).

**Fig. 2:**
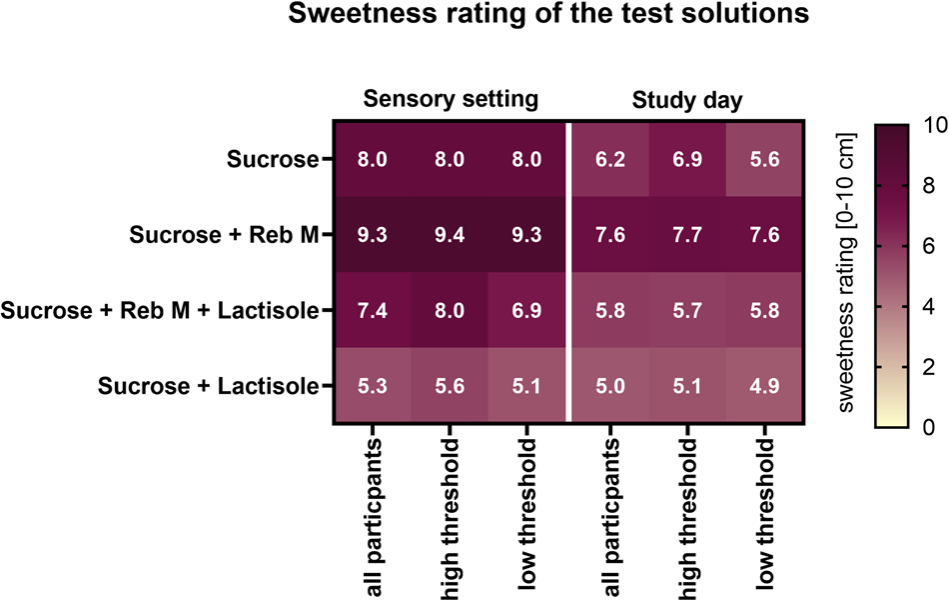
Sweetness rating of the test solutions on an unstructured scale [0–10] on the screening day in a sensory setting after receiving a scale training and on the study day. The combination of sucrose and 30 mg/L of rebaudioside M (Reb M) was rated as the sweetest solution, whereas the combination of sucrose and 15 mg/L lactisole was rated as the least sweet solution. The high sweet taste threshold group showed overall a worse ability to discriminate the different sweetness level of the test solutions.

In a first sensory evaluation, we tested whether the test persons were able to distinguish between the different sweetness levels of the test solutions after receiving a scale training. The study participants of the low sucrose detection threshold were able to discriminate between the different sweetness levels. As expected, the sucrose solution plus RebM was rated significantly sweeter than the other three test solutions. The solution containing the combination of sucrose, RebM and lactisole was rated equi-sweet to the reference test solution containing 10% sucrose, whereas the sucrose plus lactisole solution was rated least sweet. However, participants belonging to the group of the high sucrose threshold levels showed overall a poorer discrimination between the test solutions. They were only able to distinguish between the least sweet sucrose plus lactisole solution, while the other three solutions were rated equally-sweet. In addition, the test persons rated the sweetness of test solutions on each study day directly after consuming the solution. This rating was assessed without standardized reference solutions and intended to assess the individual sweetness perception of the test person directly after swallowing the test solution. In comparison to the sensory evaluation, this assessment resulted in a generally lower rating of the sweet intensity. In addition, fewer study participants were able to discriminate between the different sweetness levels without the scale training. All sensory ratings are summarized in a heatmap (Fig. 2).

### Not the perceived sweetness, but the sucrose detection threshold influenced post-load energy intake

**Fig. 3:**
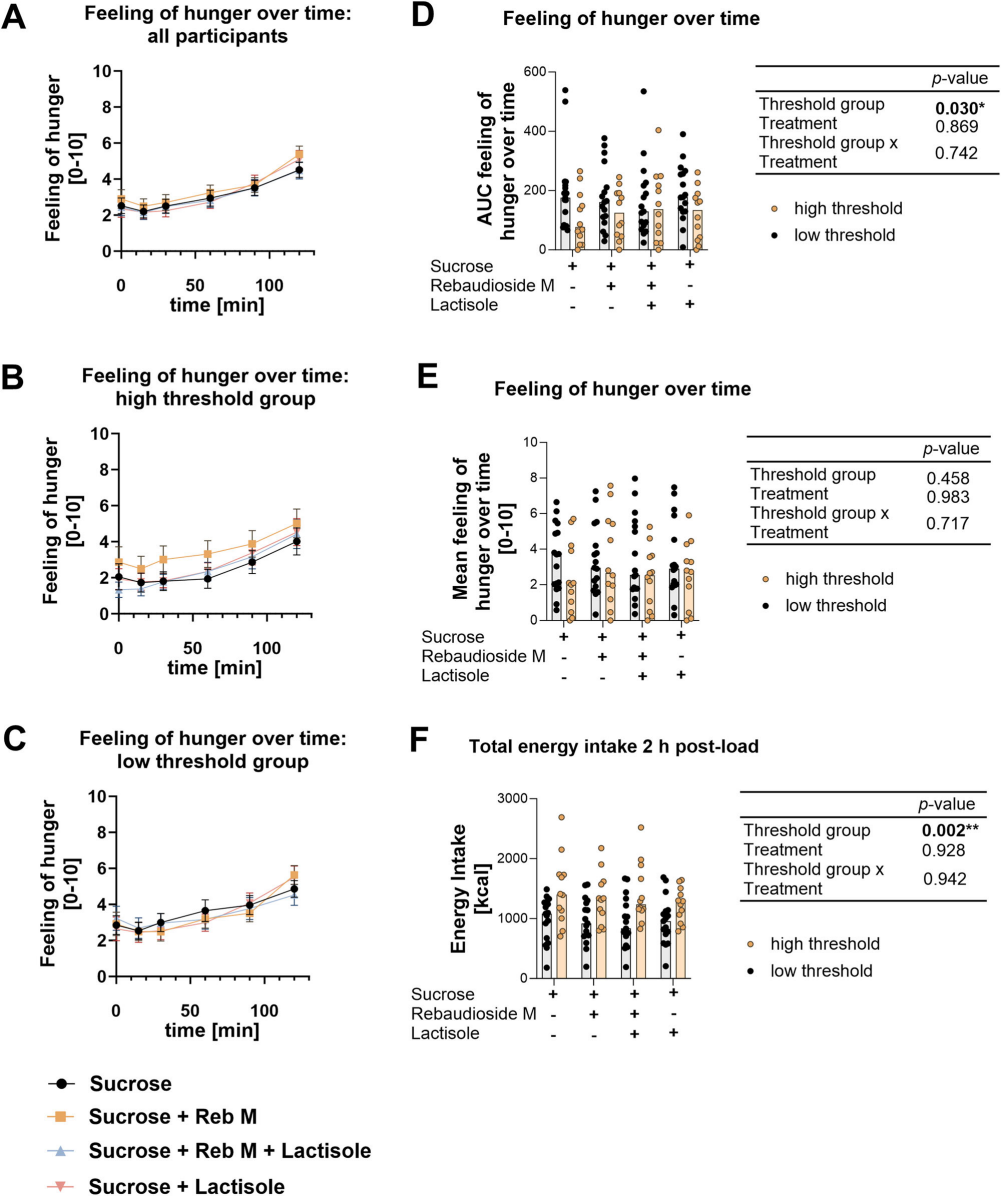
Feeling of hunger over time and post-load energy intake. The feeling of hunger, assessed at baseline and 15, 30, 60, 90, and 120 min after ingestion of the test solution on a 10 cm-visual analog scale is displayed for all participants combined (**A**), the high sucrose detection threshold group (**B**), and the low sucrose detection threshold group (**C**) separately. The feeling of hunger over time was summarized as the median AUC values (**D**) and as mean value over time (**E**). The median total energy intake from an *ad libitum* breakfast after consumption of 30 g sucrose with or without the taste modulators rebaudioside M and lactisole, or a combination thereof demonstrates an overall higher energy intake of the high threshold group (**F**). Statistically significant differences were tested by Robust Two-way ANOVA with Median Estimators; *n*_total_ = 29; *n*_low threshold_ = 17 and *n*_high threshold_ = 12. The individual responses of individual test persons are represented by circles.

The mean hunger rating over time is displayed in Fig. 3A–C. The total hunger rating over time expressed as median AUC, did not differ between the treatments and there was no association to the reported sweetness perception of the respective test solution. However, participants of the high sucrose threshold group reported to be less hungry than participants of the low sweet taste threshold group (*p* < 0.05, Fig. 3D). No difference was found when comparing the mean hunger rating value over time (Fig. 3E).

The total energy intake was assessed via a standardized *ad libitum* breakfast 2 h post administration of the test solutions but did not differ between the treatments (Fig. 3F). However, in contrast to the reported feelings of hunger, participants of the high threshold group consumed on average 402 ± 78.8 kcal (39 ± 21%) more energy than participants of the low sucrose threshold group, independent of the respective treatment (*p* < 0.01). In addition, in contrast to our hypothesis, there was no correlation (*p* > 0.05) between the energy intake and the reported sweetness perception of the respective test solution (Supplementary Figure 1).

### The perceived sweetness did not influence plasma glucose concentrations and associated hormones

**Fig. 4:**
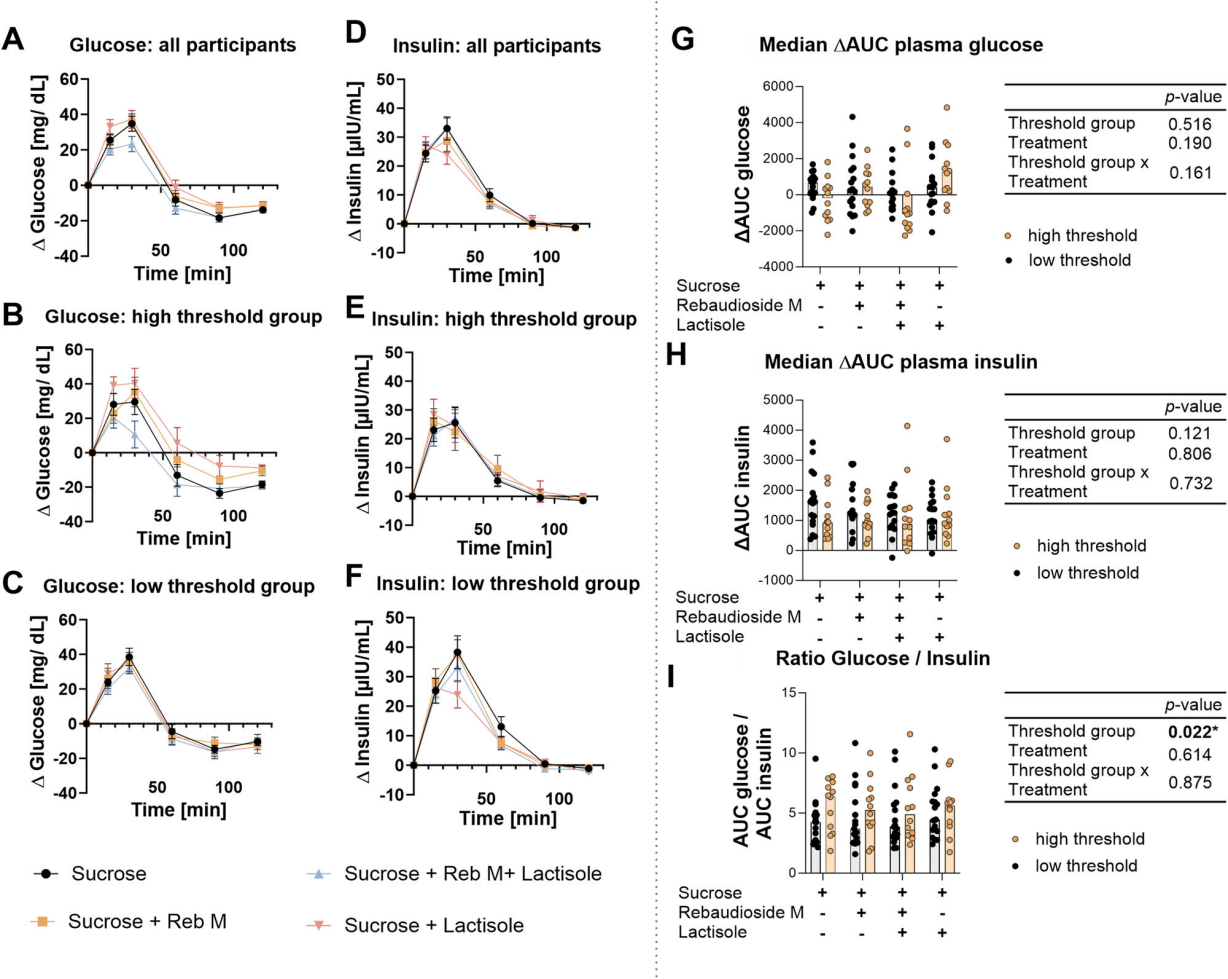
Plasma glucose and insulin concentrations over a time span of 120 min showed a regular postprandial rise and decline after consumption of 30 g sucrose. The addition of the taste modulators rebaudioside M and lactisole, or a combination thereof to the sucrose solution did not change the responses, although the participants of the high threshold group showed a stronger variation in the response. The figures illustrate the mean plasma glucose concentration over a time span of 120 min of all participants (**A**), participants of the high sweet taste threshold group (**B**), and participants of the low sweet taste threshold group (**C**). **D**–**F** show similarly the plasma insulin concentrations over time. The median AUC plasma glucose (**G**), median AUC plasma insulin (**H**), and glucose/insulin ratio (**I**) for the high and low sweet taste threshold group demonstrates a slightly higher median glucose/insulin ratio in participants of the high sweet taste threshold group, independent of the test solution. Statistical differences were tested by a Robust Two-way ANOVA with median estimators (*n*_total_ = 29; *n*_low threshold_ = 17 and *n*_high threshold_ = 12). The individual responses of the test person are represented by circles.

To analyze markers of blood glucose regulation and satiety, plasma concentration of glucose and insulin and its antagonist glucagon, as well as the incretin hormones GIP and GLP-1, and the satiety marker serotonin were analyzed. A regular time-dependent regulation of blood glucose and insulin occurred after ingestion of 30 g sucrose (Fig. 4A–F). The addition of the taste modulators RebM and lactisole, or a combination thereof, to the sucrose solution did not change the responses, although the participants of the high threshold group showed a stronger variation in the glucose response to the test solutions. The total increase of plasma glucose (Fig. 4G) and insulin (Fig. 4H) concentrations, expressed as AUC, neither differed between the treatments, nor the threshold groups. The high sucrose threshold group had overall a higher glucose/insulin ratio (*p* < 0.05) compared to the low sucrose threshold group independent of the applied test solution (Fig. 4I), without differences in the homeostatic model assessment- insulin resistance (HOMA-IR) or quantitative insulin sensitivity check index (QUICKI) between the two threshold groups (*p* > 0.05, Table 1).

**Table 1:** Characteristics of the study participants.

	Low threshold group	High threshold group	*p*-values
*n*	17	12	
Sex	Male	Male	
Age [years]	28 ± 4	26 ± 8	0.98
Height [cm]	178.2 ± 7.3	170.8 ± 42.9	0.20
Weight [kg]	74.5 ± 7.0	72.9 ± 19.7	0.42
BMI [kg/m^2^]	23.7 ± 1.8	22.3 ± 5.9	0.95
Body fat [%]	17.34 ± 8.5	13.16 ± 7.1	0.06
RMR [kcal/day]	1620.92 ± 178.2	1763.08 ± 275.7	0.08
RMR [kcal/FFM]	26.76 ± 0.4	26.44 ± 0.4	0.06
Plasma leptin [pg/ml]	4682.32 ± 2680.7	3281.69 ± 1802.6	0.13
Quicki Index	0.34 ± 0.02	0.35 ± 0.02	0.74
HOMA-IR	2.26 ± 0.83	2.17 ± 0.65	0.75

Data are presented as means ± SD. Statistical differences between threshold groups were tested using unpaired, two-tailed Student’s *t*-test.

**Fig. 5:**
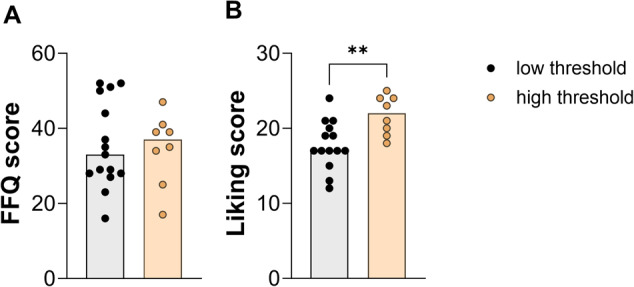
Comparison of the consumption and liking of sweet tasting foods between the two threshold groups. Displayed is the food frequency questionnaire (FFQ) score for the consumption (**A**) and the liking score (**B**) of sweet tasting foods. Statistical differences between the threshold groups were tested using an unpaired, two-tailed Student’s *t*-test (**p < 0.05), (*n*_low threshold_ = 14 and *n*_high threshold_ = 8). The individual values of the test person are represented by circles.

Dietary habits related to sweet tasting foods were assessed by means of a food frequency questionnaire (FFQ). There was no significant difference between the threshold groups (Fig. 5A). But the liking of sweet tasting food reported by test persons of the high threshold group resulted a higher liking score than for test persons of the low threshold group (*p* < 0.01, Fig. 5B).

*BMI* body mass index, *RMR* resting metabolic rate, *FFM* fat free mass, *QUICKI* quantitative insulin sensitivity check index, *HOMA-IR* homeostatic model assessment- insulin resistance.

The incretin hormones GLP-1 and GIP showed a regular postprandial rise and decline after consumption of 30 g sucrose (Supplementary Figure 2) but did overall not differ between the treatments. Similarly, plasma glucagon and serotonin concentrations did not differ between the treatments (Supplementary Figure 3).

Although no difference between the treatments was revealed, the incretin hormone GIP differed between the threshold groups; the low sucrose threshold group had overall higher GIP plasma levels compared to the high sucrose threshold group (*p* < 0.05, Supplementary Figure 4D), which might be associated with the differences in glucose/ insulin ratio. However, the response to sucrose in the GIP concentration after normalization to the baseline did not reveal differences between the two threshold groups (Supplementary Figure 2G). No difference in the overall concentrations (Supplementary Figure 4C, E, F) or the baseline-normalized responses to sucrose (Supplementary Figure 2G and Supplementary Figure 3G, H) between the threshold groups for GLP-1, glucagon, and serotonin was observed (*p* > 0.05).

To determine associations between the sweetness perception, blood glucose and satiety markers, correlation analyses were performed. In contrast to our hypothesis, there was no association (Pearson Product Moment Correlation, *p* > 0.05) between the perceived sweetness of the test solution, and the glucose, insulin, GLP-1, GIP, glucagon and serotonin concentrations in the plasma (Supplementary Figure 1).

### Participants with high and low sucrose detection threshold differ in their sweet taste liking

Participants of the low and high sucrose threshold group had a similar BMI (Table 1). However, the percent body fat (Table 1) showed a trend (*p* = 0.06) to be higher by 27.7 ± 11.2% in the low-threshold group (18.5 ± 7.4% body fat) than in the high sweet taste threshold group (13.2 ± 7.1% body fat). This was associated with trend for a 9.2 ± 4.9% higher resting metabolic rate per day (*p* = 0.08, Table 1), and a higher RMR/FFM (*p* = 0.06, Table 1) of the high threshold group compared to the low threshold group.

The adipokine hormone leptin, which is involved in satiety regulation and known to affect sweet taste perception^33^, showed no significant difference between the low and high sucrose threshold (*p* > 0.05, Table 1).

## Discussion

As the impact of the sweetness of a compound on metabolic processes is still under debate^40^, we hypothesized that individuals with a higher sensitivity towards sweet taste stimuli show a stronger hormone response when receiving a sweeter test solution compared to a less sweet test solution. The impact of sweet taste perception was addressed in two ways: on the one hand, the sweetness of a 10% sucrose solution was modulated using a sweet taste increasing (RebM) and a sweet taste inhibiting (lactisole) compound without changing its caloric load. On the other hand, as it is also discussed that the individuals’ sweet taste threshold acts as a modulator for dietary intake^41^, we considered the sucrose detection threshold as well.

In contrast to our hypothesis, the modulation of the sweetness of the 10% sucrose solution with RebM and lactisole had no impact on the subjective reported feelings of hunger over time. In addition, there was no association with the perceived sweetness of the solution. Confirming the results of the hunger rating, the total energy intake from an *ad libitum* breakfast served 120 min after ingestion of the test solution was similar after ingestion of the different test solutions. In addition, there was no association between the perceived sweetness intensity of the test solution (neither in the sensory setting nor on the study day) and the energy intake. In a previous study of our own group, the reduction of the sweetness of an equi-caloric glucose and sucrose solution had no impact on the appetite ratings^32^. In that study, the application of twice the amount of lactisole than administered in the present study resulted in a lower energy intake when applied in combination with sucrose, but not glucose. This led to the conclusion of an interplay of glucose transporters and the sweet taste receptor to regulate energy intake, which is more pronounced if less glucose is present^32^. The data of the present study showed that lower amounts of lactisole do not interfere with the regulation of energy intake. This supports the hypothesis that the metabolic effects of ingested lactisole depends on blocking extraoral sweet taste receptors and not on its antagonistic activity on oral TAS1Rs, which is responsible for the reduced sweetness perceived from the sucrose solution to which lactisole was added. Although the sweetness intensity of the test solution did not influence the reported feelings of hunger and energy intake, subjects with a higher sucrose detection threshold had an overall higher energy intake than the test persons of the low threshold group. However, no association to the sweetness rating of the test solution was found. Han et al. reported that participants with a lower sensitivity towards sweet taste ate 6% more sweet foods and 7% more carbohydrates from a buffet meal than participants in the high sensitivity group after a sweet soup preload^42^. However, it should be noted that Han et al. used a single sucrose concentration of 9 mM to divide test persons into high and low sensitivity groups. In contrast, a review based on 17 human intervention trials concluded that there is no association between the sweet threshold and energy intake, and that hedonic measurements were more likely to be associated with energy intake^41^. The discrepancy in the results and our original hypothesis may be due to different study populations. In the present study, the test persons of the high threshold group tended to have a higher fat free mass. The fat free mass is considered the main determinant of RMR, accounting for 70–80% of the total RMR^43^. Previous studies found that the fat-free mass and the RMR are both positively associated with energy intake (summarized by Hopkins et al.)^44^. We thus cannot exclude that the difference in the body composition had a stronger impact on the food intake than the sweet taste sensitivity. A lager study population is needed to assess the impact of the sweet taste sensitivity with the RMR as a covariate on the energy intake of healthy persons.

Margolskee et al. reported that the artificial sweetener sucralose induces the release of GLP-1 and GIP from the murine endocrine cell line GLUTag by activation of the sweet taste receptor^45^. As GLP-1 and GIP are known to enhance pancreatic insulin secretion and to suppress pancreatic glucagon secretion^46^, this suggests that the sweetness of a compound or compound solution—targeting the sweet taste receptor—might play a role in the regulation of blood glucose homeostasis. To analyze how the sweetness perception of humans affects these metabolic functions, we investigated markers associated with blood glucose regulation and satiety over a period of 120 min post-administration of the four different test solutions. In contrast to our hypothesis, the plasma glucose and insulin concentrations did not differ between the four tested sweet solutions and no difference between the low and the high sucrose threshold group for both parameters was found. The perceived sweetness did not impact insulin and glucose plasma concentrations following the ingestion of 30 g sucrose. However, the calculated plasma glucose/insulin ratio was higher in the high threshold group, suggesting a better post-prandial glucose response in the high sweet taste threshold group. As a previous study in diabetic persons associated the treatment with a GLP-1 receptor agonist with lower sweet taste thresholds^47^, we expected that GLP-1 might be differentially regulated between high and low sweet taste sensitive test persons. But GLP-1 concentrations over time were not different between the two threshold groups and there was no effect of the different sweet tasting treatments on GLP-1 release. Thus, the incretin hormone GLP-1 does not provide an explanation for the difference in insulin sensitivity between the threshold groups. Participants of the high threshold group had significantly lower GIP concentrations compared to the low threshold group, despite there was no difference between the treatments. A lower GIP concentration in response to sucrose may lead to lower insulin release and is associated with insulin sensitivity^48^. However, in response to sucrose, the AUC after normalization for the starting GIP-concentration was not different. Also here, the lower body fat content of persons belonging to the high-threshold group is likely to play a role for the differences in plasma GIP concentrations and may interact with sweet taste sensitivity. Although well-known for its effect on pancreatic insulin release, the effect of GIP in the regulation of satiety is less well established. But an earlier study by Raben et al. demonstrated that GIP has been negatively correlated with fullness and positively correlated with prospective food consumption, indicating an increase in feelings of hunger^49^. This is in accordance with our results, as the high threshold group had a higher energy intake and lower GIP concentrations. On the other hand, a recent study in healthy male and female participants saw lower GIP levels after the administration of resistant starch wheat compared to the ingestion of regular wheat with no difference on the self-reported perceptions of satiety^50^. Thus, the role of GIP in the regulation of food intake and a possible connection to the sweet taste sensitivity needs to be investigated in future studies.

Glucagon, which plays not only a role in glucose metabolism, but also acts on satiety by suppression of appetite^51,52^, did not show any difference between the threshold groups as well as between the different treatments. It needs to be taken into consideration that the amount of carbohydrates administered in the present study was lower than the concentration usually used for an oGTT, which may lead to smaller fluctuations in plasma glucagon concentrations. Still, as there was no difference between the treatments and no association to the perceived sweetness, a major impact of the sweetness alone can be excluded. We therefore conclude that the ingested amount of carbohydrate plays a stronger role for glucagon regulation than the activation of oral and extra-oral TAS1Rs.

There is some evidence suggesting that serotonin may have influence on glucose metabolism, towards blood glucose lowering effects^53,54^ mediated via the 5-HT_2A_ receptor^55^. We did not see any difference in serotonin levels between the treatments, matching the fact that there was also no difference in plasma glucose levels. Interestingly, an earlier study described that human taste thresholds are modulated, *inter alia*, by serotonin^56^. As we did not see any difference in serotonin plasma level between the threshold groups, we cannot confirm the results by Heath et al.^56^ in our study, which might be due to the limited number of participants of the previously reported study. A more recent study found no association between saliva serotonin levels and the sweet taste threshold in adolescents with diabetes^57^, whereas we excluded diabetic persons from our study.

The hormone leptin was originally recognized as a satiety factor and is now implicated in a wide variety of biological functions, including the regulation of glucose homeostasis^58^. The results of our human intervention study did not show any correlation of leptin and glucose, and glucose regulating hormones. Further, there was no difference between both threshold groups regarding leptin plasma levels.

To summarize, the data from the present study do not support an effect of the perceived sweet taste intensity on energy intake and blood glucose regulation, but the sucrose detection threshold might be an influencing factor. However, we were not able to demonstrate a higher hormone response in test persons with a higher sensitivity to sweet taste. This might be due the difference in the body composition between the two threshold groups, which needs further attention. We found that the test persons of the high sweet taste threshold group tended to have a lower body fat and higher resting metabolic rate at a similar BMI. The body fat mass was negatively correlated with the physical activity in previous studies^59,60^. However, we did not directly assess the physical activity in our study. Nonetheless, a higher RMR is associated with a higher energy intake^44^, which is likely to be determinant factor of the difference in energy intake in our study as the post-prandial hormone response was not different between the two threshold groups. In addition, the test persons of the high threshold group reported a higher liking for sweet tasting foods. The connection between the sweet taste sensitivity, sweet liking, and body composition may therefore be a stronger influencing factor of the regulation of blood glucose homeostasis and energy intake than the perceived sweetness of the carbohydrate-containing solution. Here, also the plasticity of the gustatory system in response to dietary and lifestyle associated factors including changes on the gene expression level of taste markers may be taken into consideration^61^. Feeney et al. concluded that habitual exercisers are more sensitive to sweet taste perception compared to inactive study participants, based on the perceived intensity of a sucrose test solution^62^. An early study reported that the detection threshold for sucrose solutions decreased after a half-marathon^63^, which also supports a strong association with the physical activity and sweet taste sensitivity. In addition to sweet taste sensitivity, sweet taste liking may be associated with body composition. This was investigated only recently by Iatridi et al., who described two studies with 274 participants and 148 participants respectively, to investigate whether sweet liking is related to body composition^64^. They concluded that fat free mass (FFM) is the main anthropometric compartment most strongly associated with sweet liking^64^, which is in accordance to the results of the present study. Therefore, we hypothesize that the physical activity level increases liking for sweet taste and decreases sweet taste sensitivity. Although participants of the high sucrose threshold group liked sweet tasting foods significantly more than participants with a low sucrose threshold, the low and the high threshold group did not differ between the self-reported dietary habits regarding sweet tasting foods. In summary, our results support an interplay of sucrose detection thresholds, liking of sweet tasting foods, and the body composition at the same BMI. Future studies need to demonstrate whether the sucrose detection threshold is associated with a higher abundance of TAS1Rs in the oral cavity as well as in the gastrointestinal tract.

Our study has potential limitations. First, no female study participants were included in this study due to natural fluctuations in blood glucose regulation. Sex-specific effects thus need to be addressed in larger human intervention trials in the future. Moreover, no detailed questionnaire about the participants’ physical activities was included, based on the results of the current trial, this should be considered for future studies as well. In addition, we only included healthy volunteers, the study results cannot be extrapolated to persons with impaired glucose tolerance.

In conclusion, we demonstrated here that the sweetness level and the perceived sweetness of isocaloric sucrose solutions did not influence post-load energy intake and blood glucose concentrations, as well as regulating hormones in healthy males. However, test persons belonging to the high sucrose detection threshold group had a higher energy intake and better post-prandial glucose response than the test persons of the low threshold group, combined with a higher liking for sweet-tasting food. The assumed increased physical activity level in the high threshold group may have influenced energy intake and insulin sensitivity and an association of the physical activity level and the sucrose detection threshold needs to be confirmed in future studies.

## Methods

### Study design

The study was designed as a single blinded, cross-over human intervention study. Participants received four different interventions on four separate study days, with each intervention carried out at least 7 days apart. The study design was approved by the ethical committee of the University of Vienna (approval no. 00568, and addendum no. 00583), and registered at ClinicalTrials.gov (ID NCT04991714). All volunteers signed a written informed consent and data privacy guidelines prior to the intervention.

Participants were randomly assigned to treatments using the online tool “randomizer.org” and were not given any information about order of treatments (single blinded). To confirm the good state of health of the participants, a medical screening was conducted prior to the first intervention. A standard oral glucose tolerance test (oGTT) with 75 g glucose in 300 mL of water was performed to ensure physiological response to glucose consumption. Urine and blood glucose concentrations were measured before 60 and 120 min post oGTT. A compliant elevation of blood glucose levels during oGTT was additionally monitored at 0, 15, and 30 min with a blood glucose meter in the capillary of the fingertip (Accu-Chek Performa, Roche, Switzerland). Fasting hematological parameters and plasma lipids were analyzed by a local medical diagnostics laboratory (“Ihr Labor 1220”, Dr. Gabriele Greiner, Vienna, Austria). Systolic and diastolic blood pressure were analyzed in triplicate using an upper arm blood pressure monitor (Medisana, Germany). Basic anthropometric measurements were recorded, namely body height, with a precision of 0.1 cm by means of a stadiometer (Seca, Germany), and body weight to the nearest of 0.1 kg using a body scale (Soehnle, Germany). In addition, the body fat, fat free mass, and resting metabolic rate (RMR) was determined using a Bod Pod ® (COSMED Italy (HQ) | USA | United Kingdom | Germany | France | Denmark | Switzerland | The Netherlands | Australia | Hong Kong). Participants were asked to fill out a SCOFF questionnaire, to exclude eating disorders^65^, and a screening questionnaire including questions such as food allergies or intolerances, chronic diseases and basic health information.

Power analysis by means of the software GPower 3.1^66^ resulted in an estimated number of 30 test subjects based on a previous study^24^, with an effect size of 0.51 (power of 0.85, alpha = 0.05). A total of 35 subjects was recruited, out of which 30 volunteers passed the medical screening. One volunteer did not finish the study due to an inability to fulfill the COVID-19 hygiene regulations that became valid after the start of the study. Accordingly, 29 participants completed all four treatments and were included in the study. The mean characteristics of the participants are given in Table 1.

An overview of the study design is depicted in Fig. 1. On each study day, fasting blood glucose levels were determined via finger-prick blood test and a first blood sample (t0) after 12 h overnight fast was collected via a peripheral IV catheter (Venflon, BD). The participants were then asked to drink the test solution within 5 min and to rate the perceived sweetness of the respective test solution on a 10 cm unstructured line scale, from “not at all” (0 cm) to “very intensive” (10 cm) sweetness. This sweetness rating was conducted without a previous scale training. Further blood samples were collected 15, 30, 60, 90, and 120 min after drinking of the test solution. At each blood collection time point, the participants’ feeling of hunger was assessed using a 10 cm visual analogue scale (VAS) (see below for more details). After the last blood collection (t120), a standard continental breakfast was served to determine energy intake as described previously^32^.

### Test solutions

A concentration of 10% (w/v) sucrose in 300 mL water was chosen as a reference solution, according to amount of sugar commonly found in soft drinks and juices. The sweetness of the sucrose-based solution was then modulated using the steviol glycoside RebM, or the sweet taste inhibitor lactisole, respectively. The following four different test solutions (TS) were tested accordingly:

TS1: 30 g sucrose in 300 mL water (reference solution),

TS2: 30 g sucrose + 18 mg RebM in 300 mL water (sweeter than TS1),

TS3: 30 g sucrose + 18 mg RebM + 9 mg lactisole in 300 mL water (equi-sweet to TS1),

TS4: 30 g sucrose + 9 mg lactisole in 300 mL water (less sweet than TS1).

### Sensory evaluation

On the screening day, the volunteers’ sucrose detection threshold was determined according to DIN EN ISO 3972:2013-12 as described previously^24^. In brief, the volunteers received 20 mL of nine ascending concentrations of sucrose dissolved in tap water ranging from 0.32 to 20 g/L. The threshold value reported here refers to the test concentration at which the test solution was correctly recognized as “sweet” for the first time. The sweet intensity of each test solution was rated on an unstructured scale [0–10 cm] after a scale-training with five concentrations ranging from 0 to 100 g/L sucrose as “not at all” to “very intensive” sweetness. Each test solution (20 mL) for the sensory evaluation was provided in a 40 mL plastic beaker with a three-figures random code. All tests were carried out in sip-and-spit mode and the participants were advised to neutralize with tap water in between the tests. On the study days, the sweet intensity was rated without a previous scale-training right after consumption of the total amount of 300 mL. All test solutions were dissolved in tap water.

### Study participants

The interventional part of the human study was conducted between July 2021 and December 2021 at the University of Vienna following the principles of the declaration of Helsinki. Thirty-five male volunteers were recruited using web advertising. Eligible for study participation were metabolic healthy males aged between 18 and 45 years with a body mass index between 18.5–29.9 kg/m^2^ and no taste disorders. Exclusion criteria were fasting blood glucose >120 mg/dl^1^ and major chronic diseases, metabolic diseases such as T2DM or lipometabolic disorders, tobacco consumption, medical treatment, alcohol or drug abuse as well as intolerances or allergies to test products. Women were excluded of the study due to hormonal variations based on menstrual cycle^67^. Based on their sucrose detection threshold determined as described above, the study participants were divided into two threshold groups, the “low sweet taste threshold” group (0.55–4.32 g/L) and the “high sweet taste threshold” group (7.2–20 g/L). The cut-off value of 4.32 g/L sucrose for the groups was chosen according to previous studies that identified average recognition concentrations in comparable untrained populations at 4.4 g/L or below^68,69^. The study population characteristics of the present study are provided in Table 1.

### Analysis of plasma concentration of glucose, insulin, GLP-1, GIP, glucagon, serotonin, and leptin

For the quantitative analysis of GLP-1, GIP, glucagon, serotonin, and leptin plasma concentrations, venous blood samples were collected in EDTA-coated monovettes (Sarstedt, Germany), centrifuged immediately at 1800 × *g* at 4 °C for 15 min. The plasma samples were stored at −80 °C until further analysis. Samples for the determination of serotonin were additionally centrifuged for 1 min at 7000 × *g* at 4 °C to remove blood platelets. To determine plasma glucose concentrations, blood was collected in fluoride-coated monovettes, and for insulin, heparin-coated monovettes (both Sarstedt, Germany) were used as described previously^24,32^.

Plasma glucose concentrations were quantified by a colorimetric assay (Cayman Europe, Estonia). Insulin (Iason, Austria), GLP-1 (Merck Millipore, Germany), GIP (RayBiotech, USA), glucagon (Thermo-Fisher, USA), and leptin (abcam, UK) concentrations in the plasma were assessed using sandwich ELISA, respectively. Plasma serotonin was assessed by means of competitive ELISA (DLD Diagnostic, Germany). All assays were performed according to manufacturer’s protocol.

### Participant’s rating of hunger

Test subjects completed a visual analogue scale (VAS), thereby reporting their subjective feeling of hunger at each blood collection time points (0, 15, 30, 60, 90, and 120 min). The VAS was designed as a 10 cm unstructured scale, starting at 0 for “not hungry at all” to 10 cm “extremely hungry”.

### Total energy intake

After the last blood collection time point total energy intake from standardized, continental, *ad libitum* breakfast typical for the Austrian population was determined. The total energy content of the breakfast was about 11.3 MJ, with a proportion of 46% carbohydrate, 41% fat, and 13% protein. In more detail, the breakfast consisted of four rolls, three slices of bread, 80 g of butter, 60 g of honey, 100 g of strawberry jam, 6 slices cheese (~125 g), 5 slices ham (~100 g), 180 g of wild berry yoghurt, 200 mL coffee or tea, 20 g of sugar, 40 g of coffee creamer, and 200 mL of water. For participants following a vegetarian or vegan diet, isocaloric plant-based alternatives were provided. Quantitative energy consumption was assessed by back weighing the food that was not consumed. Calculation of energy and nutrient intake was performed using the software nut.s v1.32.50 (nutritional.software, Vienna, Austria).

### Food frequency questionnaire and liking questionnaire

An online questionnaire to determine the consumption and preference of sweet tasting food was sent out to the study participants. The questionnaire was divided into two categories: (1) Food frequency questionnaire (FFQ), to determine how often certain sweet tasting food groups are usually consumed, and (2) liking questionnaire, to assess how much certain sweet tasting food groups are liked. Participants had eight choices to indicate the frequency of consumption of each of the 20 sweet tasting food items from “never” to “several times per day”. Sweet tasting foods were grouped into following categories: fresh fruit, dried fruits, compote or fruit puree, sugar to sweeten drinks, honey/maple/agave syrup to sweeten drinks, sweetener to sweeten drinks, chocolate, ice cream, sugar confectionery, baked confectionery, sweetened dairy products, sweetened drinks on dairy basis, sweet spreads, breakfast cereals, juice, energy drinks, soft drinks, protein shakes, sport drinks, and sweetened alcoholic beverages. The liking questionnaire consisted of seven categories: fresh fruit, chocolate, sugar confectionery, baked confectionery, sweetened dairy products, sweet beverages, and sweet spreads, with five response possibilities from “I don’t like it at all” (0 points) to “I like it very much” (4 points). The questionnaire data were analyzed by formation of an overall liking score per category, by adding up the points from corresponding foods.

### Statistical analysis

Statistical analyses were performed using the statistical computing language R (version 4.2.2)^70^. Differences between treatments or threshold groups including interaction effects were determined by means of a robust two-way ANOVA for medians followed by post hoc group comparisons using medians as estimator of location as implemented in the WRS2 extension package^71^.

In case of the time-dependent hormone regulation data, the baseline corrected, incremental area under the curve (AUC) was calculated according to the trapezoidal rule using the trapz function from caTools function^72^. For glucose and insulin, the positive ΔAUC over time, and for GLP-1, GIP, glucagon, and serotonin net ΔAUC over time was calculated. The peak levels for each of the hormones was compared by means of a robust two-way ANOVA with median estimators.

Differences of leptin and % body fat between the two threshold groups were determined by two-tailed, unpaired *t*-test. Correlation was assessed by Spearman Correlation between the sucrose detection thresholds and plasma concentrations of glucose, insulin, GLP-1, GIP, glucagon, and serotonin. Differences regarding FFQ and liking score between both threshold groups were determined by a two-tailed, unpaired *t*-test.

## Supplementary information

Supplementary Material

## Data Availability

The datasets used and/or analyzed during the current study available from the corresponding author on reasonable request.
