# Supplementary material for: A high sucrose detection threshold is associated with increased energy intake and improved post-prandial glucose response independent of the sweetness intensity of isocaloric sucrose solutions
Source: NPJ Metab Health Dis. 2024 Jan 29;2:1. doi: 10.1038/s44324-023-00003-0 (PMC12118705; doi:10.1038/s44324-023-00003-0)
Supplement: Supplementary file 1 — Supplementary Material [file 44324_2023_3_MOESM1_ESM.pdf]

Supplementary Material to:

A high sucrose detection threshold increases energy intake independent of the sweetness of sucrose solutions

Verena Preinfalk, Kerstin Schweiger, Leonie Hüller, Andreas Dunkel, Isabella Kimmeswenger, Corinna M. Deck, Petra Rust, Veronika Somoza, Gerhard E. Krammer, Jakob P. Ley & Barbara Lieder

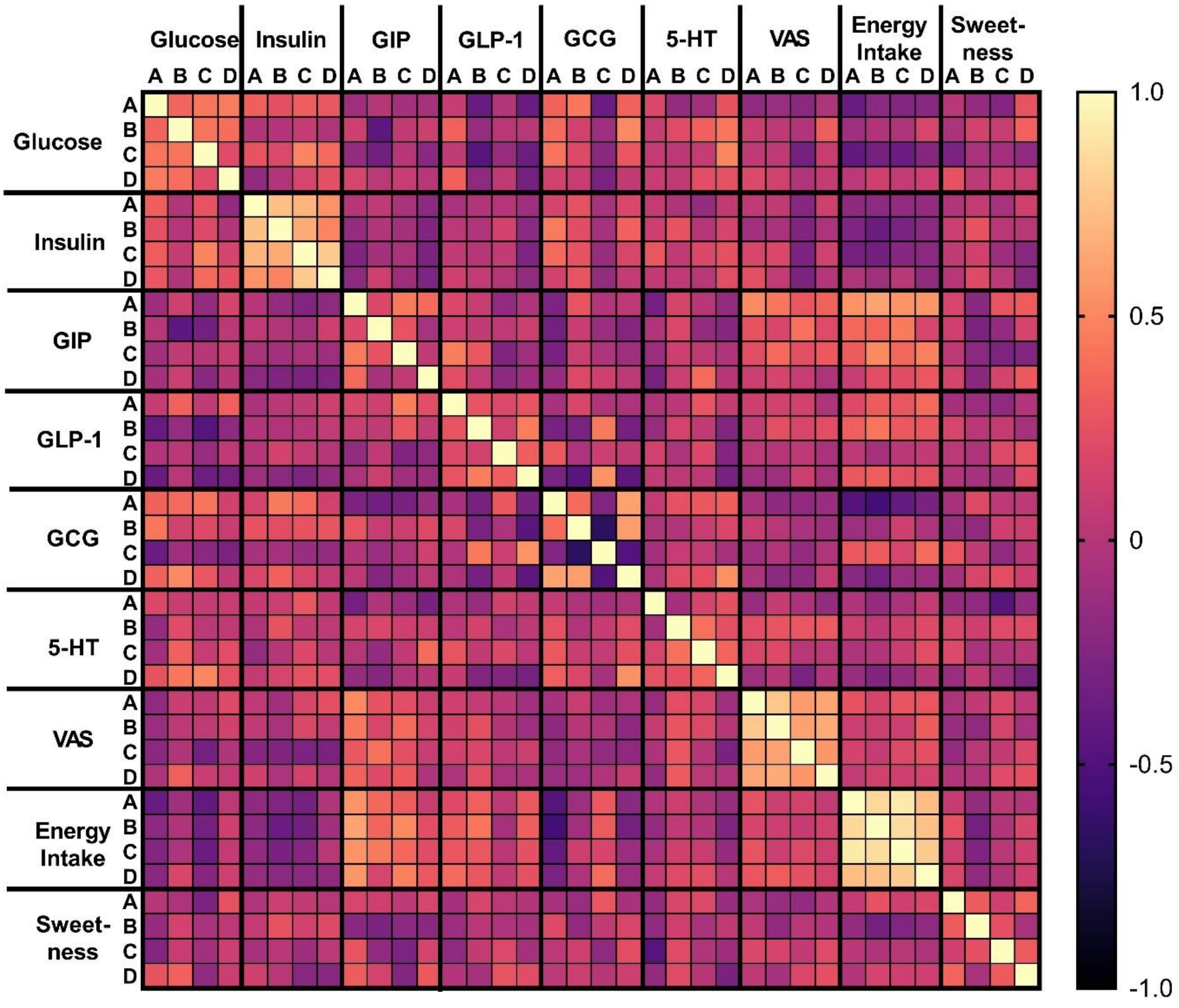

Supplementary Figure 1. Correlation Matrix of glucose, insulin, GIP, GLP-1, GCG (glucagon), 5-HT (serotonin), VAS (visual analogue scale), energy intake, and the sweetness rating of the test solution. The reported sweetness of the test solutions A-D did not correlate with the tested outcome measures (Pearson Product Moment Correlation, $p>0.05$). Test solutions A-D: **A**: sucrose; **B**: sucrose + rebaudioside M; **C**: sucrose + rebaudioside M + lactisole; **D**: sucrose + lactisole

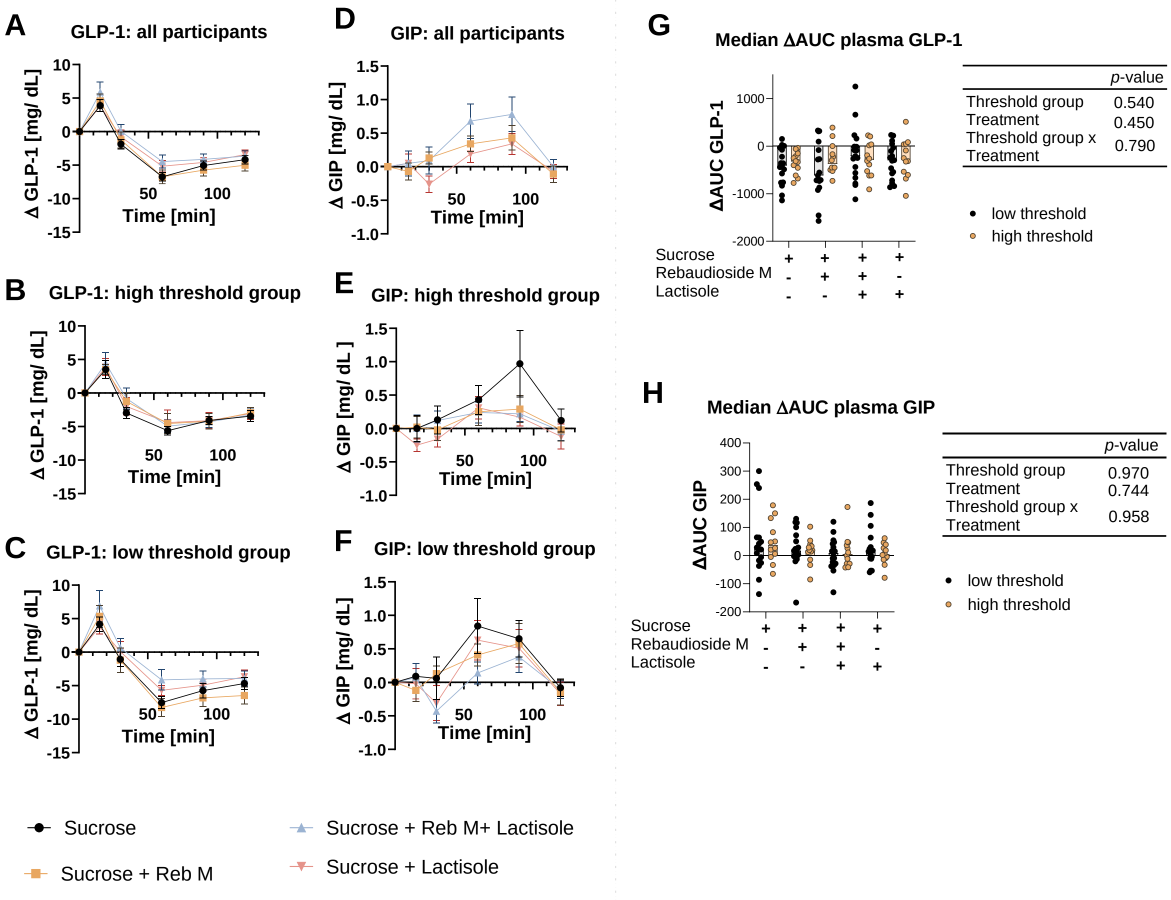

Supplementary Figure 2. Plasma concentrations of the two incretin hormones GLP-1 and GIP over a time span of 120 min show a regular postprandial rise and decline after consumption of 30 g sucrose. The addition of the taste modulators rebaudioside M and lactisole, or a combination thereof to the sucrose solution did not change the responses of GLP-1. The GIP concentrations were not differentially influenced by the different sweetness level, participants of the high threshold group had overall slightly lower GIP plasma concentrations in response to the sucrose solution. The figures illustrate the mean plasma GLP-1 concentration over a time span of 120 min of all participants (**A**), participants of the high sweet taste threshold group (**B**), and participants of the low sweet taste threshold group (**C**). Panels **D to F** show similarly the plasma GIP concentrations over time. The median AUC plasma GLP-1 (**G**), median AUC plasma GIP (**H**) for the high and low sweet taste threshold group is shown separately. Statistical differences were tested by a Robust Two-way ANOVA with median estimators (n_{total} = 29; $n_{low\ threshold}$ = 17 and $n_{high\ threshold}$ = 12). The individual responses of the test person are represented by circles.

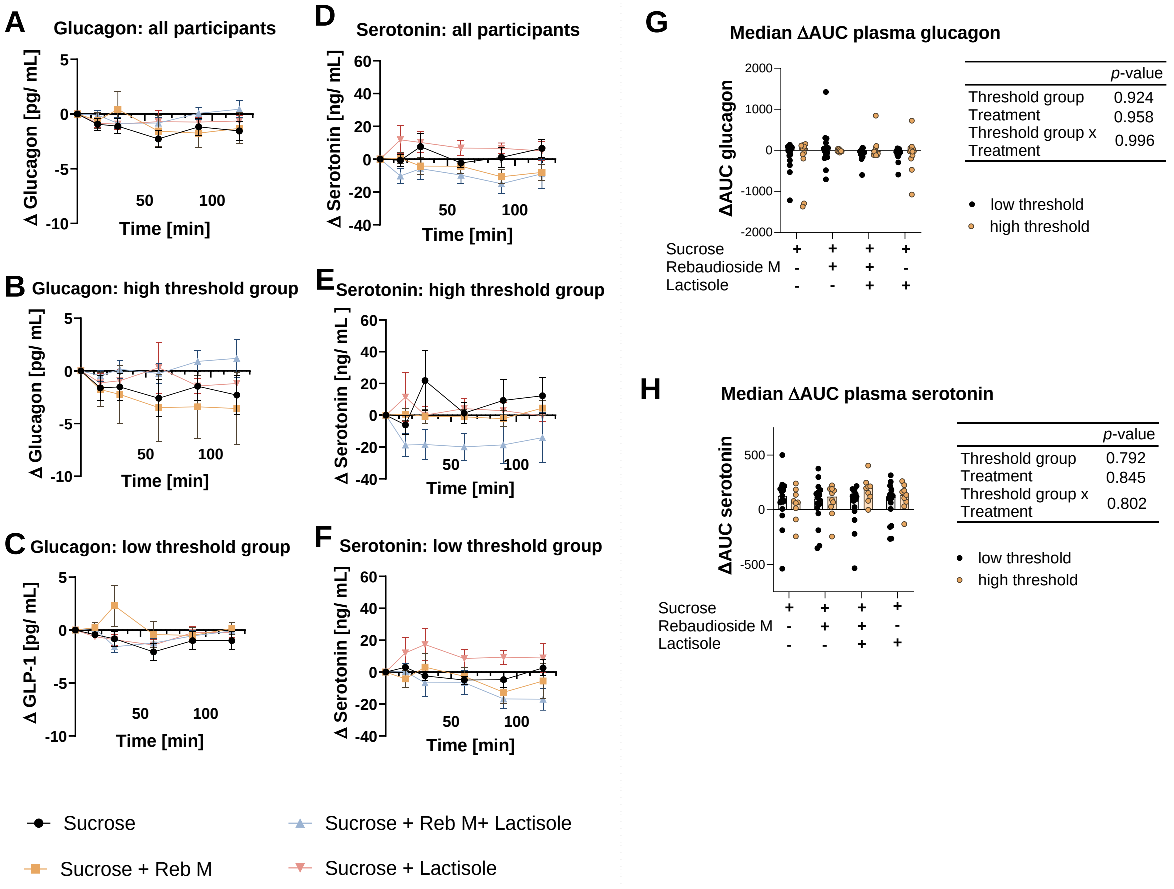

Supplementary Figure 3. Plasma glucagon and serotonin concentrations over a time span of 120 min show varying individual response to the ingestion of 30 g sucrose. The addition of the taste modulators rebaudioside M and lactisole, or a combination thereof to the sucrose solution did not change the responses, although the participants of the high threshold group showed a stronger variation in the response of both parameters. The figures illustrate the mean plasma glucagon concentration over a time span of 120 min of all participants (**A**), participants of the high sweet taste threshold group (**B**), and participants of the low sweet taste threshold group (**C**). Panels **D** to **F** show similarly the plasma serotonin concentrations over time. The median AUC plasma glucagon (**G**), and the median AUC plasma serotonin (**H**), for the high and low sweet taste threshold group also demonstrates the high individual response of the test persons, independent of the test solution or the sucrose detection threshold. Statistical differences were tested by a Robust Two-way ANOVA with median estimators (n_{total} = 29; $n_{low\ threshold}$ = 17 and $n_{high\ threshold}$ = 12). The individual responses of the test person are represented by circles.

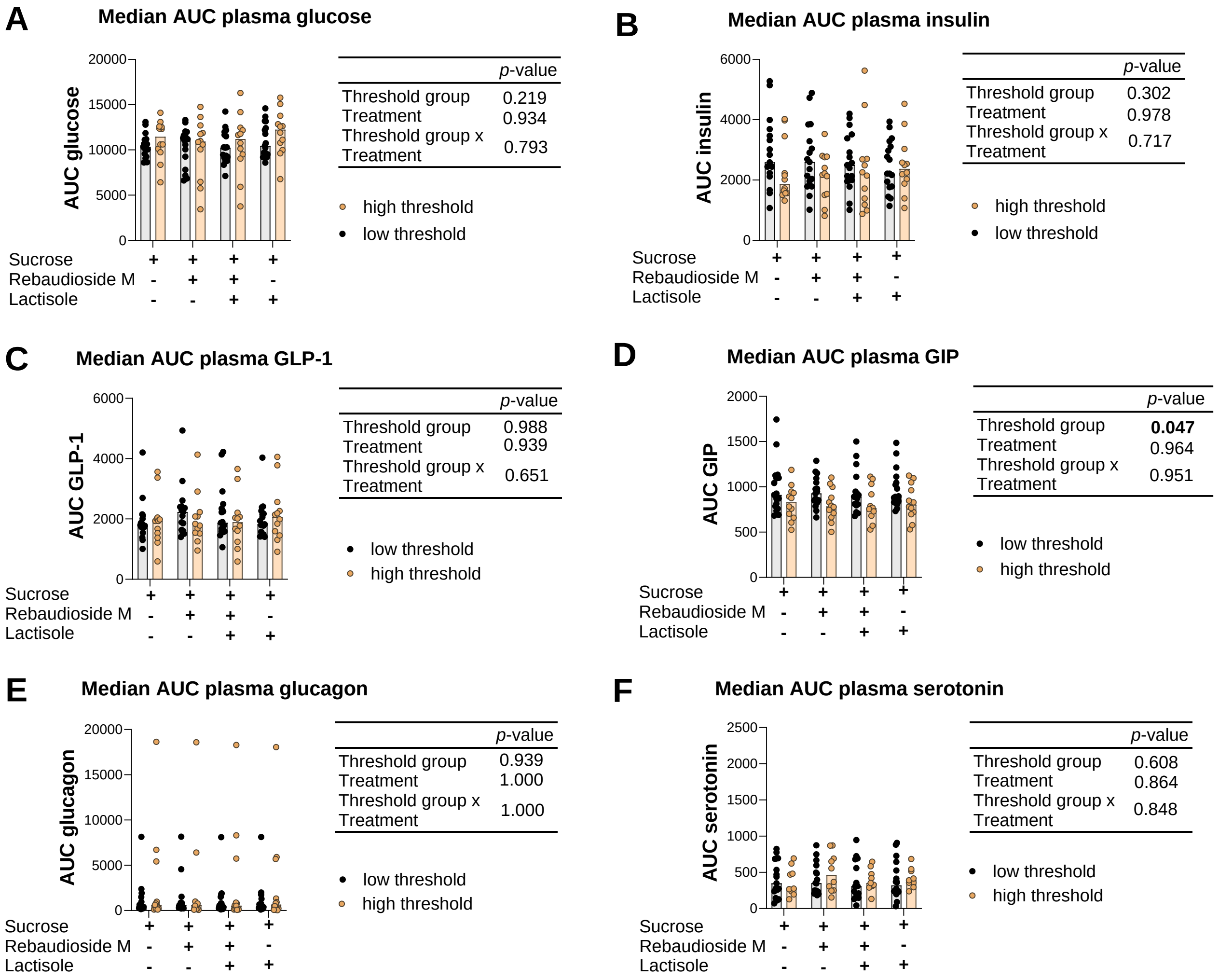

Supplementary Figure 4. Median AUC plasma glucose (**A**), median AUC plasma insulin (**B**), median AUC GLP-1 (**C**), median AUC plasma GIP (**D**), median AUC plasma glucagon (**E**), and median AUC plasma serotonin (**F**) for the high and low sweet taste threshold group demonstrates an overall higher GIP concentration in participants of the low sweet taste threshold group, independent of the test solution. Statistical differences were tested by a Robust Two-way ANOVA with median estimators (n_{total} = 29; $n_{low\ threshold}$ = 17 and $n_{high\ threshold}$ = 12). The individual responses of the test person are represented by circles.
